# Extraconal cavernous hemangioma of orbit: A case report

**DOI:** 10.4103/0971-3026.43849

**Published:** 2008-11

**Authors:** Rama Anand, Kavita Deria, Pankaj Sharma, MK. Narula, Rajiv Garg

**Affiliations:** Department of Radiodiagnosis, Lady Hardinge Medical College and Associated Hospitals, New Delhi, India

**Keywords:** Extraconal, hemangioma, orbit

## Abstract

Cavernous hemangioma is the most common benign noninfiltrative neoplasm of the orbit. Most cavernous hemangiomas are intraconal and lateral in location. We present a case of a cavernous hemangioma with an unusual extraconal and superomedial location.

Cavernous hemangioma is the most common benign noninfiltrative neoplasm of the orbit. Most cavernous hemangiomas are intraconal and lateral in location. They result from the new formation of vessels, proliferation of tissue components of the vessel wall, and hyperplasia of cellular elements ordinarily concerned with the genesis of vascular tissue.[1] A case of cavernous hemangioma with an unusual extraconal superomedial location is presented.

## Case Report

An 18-year-old woman presented with gradually progressive proptosis of the left eye with swelling of the superomedial quadrant of the left orbit. The swelling did not change in size with the Valsalva maneuver, coughing, straining, or change in head position. The cornea and sclera were normal.

**Figure 1:**
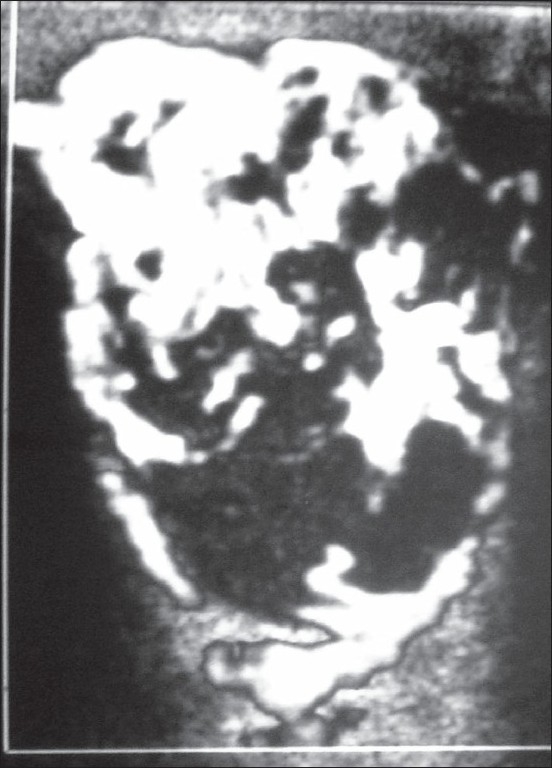
Color Doppler shows an extraconal mass (arrow) with rich vascularity in the superomedial quadrant

**Figure 2:**
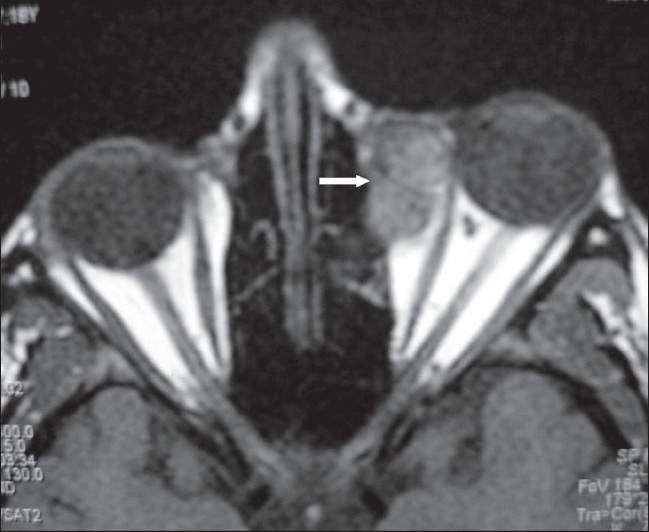
Axial T1W MRI image shows a well-defined extraconal lesion (arrow), isointense to the extraocular muscles, displacing the medial rectus laterally, anteriorly

**Figure 3:**
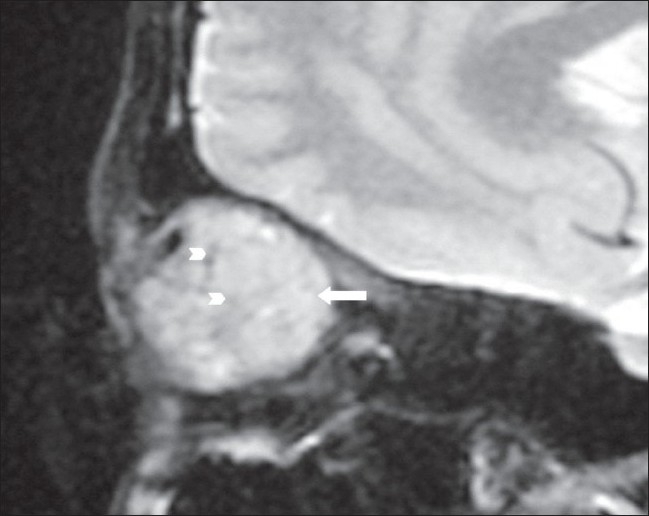
Sagittal T2W MRI image shows a hyperintense lesion (arrow) with signal voids (arrowheads) within

**Figure 4 (A, B):**
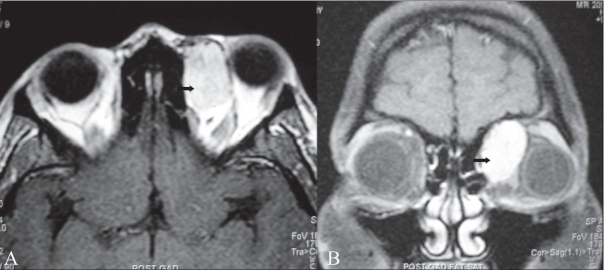
Post-contrast axial (A) and coronal (B) T1W MRI images shows intense enhancement of the lesion (arrows). Inferolateral displacement of the globe is well seen on the coronal image (B)

USG revealed a well-encapsulated, compressible, echogenic mass lesion (approximately 2.8 × 1.7 cm in size) situated superomedially in the extraconal space of the left orbit. On Doppler, the mass showed multiple vascular channels (both arterial and venous channels) suggestive of a vascular lesion [Figure 1]. Small low-flow feeding arteries were seen. MRI showed an oval, encapsulated, superomedial, and extraconal mass in the left orbit. The mass appeared isointense to orbital muscle on T1W [Figure 2] and mildly hyperintense to orbital muscle on T2W [Figure 3] images, with intense enhancement on post-gadolinium scans [Figure 4a and b]. There was lateral displacement of the medial rectus muscle anteriorly with inferolateral displacement of the globe. The intraocular muscles and optic nerve sheath complex showed normal signal intensity.

The tumor was excised using an anterior orbitotomy approach. Histopathology confirmed the diagnosis of a cavernous hemangioma.

## Discussion

Hemangiomas (benign vascular neoplasms) are classified as capillary and cavernous. A capillary hemangioma usually presents in the first year of life and often increases in size for 6–10 months before slowly involuting. Cavernous hemangiomas are the most common benign noninfiltrative neoplasms of the orbit and have a slowly progressive mass effect.[2] They are usually present in the second to fourth decades of life and are more frequent in females.[3,4]

A slowly progressive proptosis is the typical presenting symptom. Extraocular muscle impairment and impaired visual function are seen with large lesions and with lesions located at the orbital apex. Clinically, these tumors are soft and do not change in size with the Valsalva maneuver or with coughing, straining, or change in the head position.[3,4]

Most cavernous hemangiomas are typically intraconal and lateral in location. Extraconal and medial locations are uncommon.[3,5] USG, CT scan, and MRI are useful imaging techniques for the evaluation of cavernous hemangiomas. Angiography is rarely required.[5,6]

The tumors are round to oval in shape with well-defined borders and a specific ‘honeycomb’ pattern of alternating weak and strong echoes corresponding to their structure, with flow on color Doppler.[3,5] They may show a negative Doppler phenomenon which is attributable to the stagnant blood within the vascular spaces.[7]

CT scan shows discrete lesions with varying degrees of enhancement. On MRI, the lesions are homogenous, isointense to muscle on T1W images, and hyperintense to muscle on T2W images, as was seen in the present case. Variable homogenous or inhomogeneous contrast enhancement is the rule.[3,5] These lesions have a small arterial input with small venous outflow channels and very slow flow within.[6]

Histopathology reveals a fine capsule that surrounds a tumor consisting of large endothelium-lined channels with abundant, loosely distributed smooth muscle in the vascular wall and stroma.[3,6]

Treatment of the tumor is surgical excision. Complete excision is generally accomplished as the tumor is well encapsulated with relatively few feeding vessels.[6,8]
